# Correlation of frailty assessment metrics in one-year follow-up of aged care residents: a sub-study of a randomised controlled trial

**DOI:** 10.1007/s40520-023-02491-y

**Published:** 2023-07-14

**Authors:** A. Q. Andrade, I. Widagdo, R. Lim, T.-L. Kelly, G. Parfitt, N. Pratt, R. L. Bilton, E. E. Roughead

**Affiliations:** 1 UniSA Clinical and Health Sciences, University of South Australia, Adelaide, 5001 Australia; 2 UniSA Allied Health & Human Performance, University of South Australia, Adelaide, Australia; 3 College of Nursing and Health Sciences, Flinders University, Adelaide, Australia

**Keywords:** Frailty, Aged care, Cognitive frailty

## Abstract

**Introduction:**

Frailty is increasingly recognised as a dynamic syndrome, with multiple causes, dimensions and consequences. There is little understanding of how those frailty assessment metrics interact over time. The aim of this study was to describe the longitudinal correlation between five frailty metrics, namely multimorbidity, muscular strength, mood alterations, cognitive capacity, and functional capacity in a cohort study of aged care (nursing home) residents.

**Methods:**

248 aged care residents with Frailty Index at baseline of < 0.4 and no dementia were followed for 12 months. A multimorbidity score and an activity of daily living limitation score were created using individual items of the Frailty Index. Muscular strength was measured by grip strength. Cognitive capacity was measured using the Montreal Cognitive Assessment (MoCA) test. Mood alterations were measured using the anxiety/depression screening question from EQ-5D. We analysed the inter-individual correlation at baseline, association between baseline and future change, and within-individual correlation at baseline, 6 and 12 months.

**Results:**

Population analysis shows that metrics were not associated at baseline. All of the studied metrics at baseline were associated with change in 12 months, with the exception of anxiety/depression scores. Pairwise within-individual correlation was strong between MoCA and grip strength (0.13, *p* = 0.02) and activity of daily living (− 0.48, *p* < 0.001), and between activities of daily living and multimorbidity index (0.28, *p* < 0.001). No within-individual correlation was found between anxiety depression score and other metrics.

**Conclusion:**

The results suggest an interdependence between comorbidities, physical capacity, cognition and activities of daily living in aged care residents. Comprehensive measurement of frailty-related metrics may provide improved understanding of frailty progression at later life stages.

**Supplementary Information:**

The online version contains supplementary material available at 10.1007/s40520-023-02491-y.

## Introduction

Population ageing and longer life expectancies bring new challenges for geriatric care and research [1]. Ageing has a long-observed process of increased morbidity and decline in physiological capacity. This process is commonly conceptualised as frailty, defined by the WHO as “a progressive age-related decline in physiological systems that results in decreased reserves of intrinsic capacity, which confers extreme vulnerability to stressors and increases the risk of a range of adverse health outcomes” [2].

Frailty has important implications for clinical practice. As an indication of risk of poor clinical outcomes, frailty assessment can inform clinical decisions and enable avoidance of potentially harmful treatments. As an indication of decline in capacity, associated with but not dependent on diseases, frailty can potentially be stopped or even reversed with adequate treatment.

There is growing evidence that homeostatic instability is a result of the inadequate interaction between the multiple systems [3]. Age and age-related diseases are associated with capacity reduction in multiple physiological systems, or dimensions [4]. As a result, frailty can be evidenced by deficits in (1) Physical dimension, a syndrome characterised by typical biological changes associated with age usually called phenotypic frailty. It was characterised by muscle weakening and sarcopenia, reduced energy and unintentional weight loss [5]; (2) Cognitive dimension, defined as the simultaneous decline in cognitive and physical capacity in absence of dementia [6]; (3) Psychological dimension, representing the decline in mental resilience as a consequence of age-altered brain function  [7]. Finally, frailty is strongly associated with activities of daily living disability [8], which impacts on the capacity for independent living and quality of life.

Frailty is not a static syndrome, but one in which there is variable decline and interactions between frailty dimensions over time. Physiological capacity may fluctuate over time and individuals can transition from different frail states [9–12]. Physical frailty may result in future cognitive impairment and is associated with higher risk of dementia [13]. Psychosocial frailty has also been associated with future cognitive impairment [9, 10]. There is evidence that frailty accelerates in late-life [14], and it is possible that aged care residents exhibit such frailty trajectories with significant decline in health outcomes and quality of life.

Describing longitudinal frailty along the different metrics can yield a better understanding of the overall frailty syndrome and its progression in late-life. The aim of this study was to describe the longitudinal correlation between five frailty metrics, namely multimorbidity, muscular strength, mood alterations, cognitive capacity, and functional capacity, in a prospective 1-year follow up of a cohort of aged care residents.

## Methods

### Study design

This was a secondary study using data collected during the Reducing Medicine-induced Deterioration and Adverse Reactions (ReMInDAR) trial [15]. In brief, the ReMInDAR trial was a multicentre, open label, randomised controlled trial involving 39 aged care homes with a 12-month follow-up period. The intervention was an on-going pharmacist-led intervention occurring every 8 weeks over 12 months aimed at preventing medicine-induced frailty.

A total of 248 aged care residents from 39 aged care homes across two Australian states, South Australia and Tasmania, were included in the trial. Aged care homes provide a range of services for individuals unable to live independently at home, including assistance with daily living (food preparation, showering) and health care (medicine administration, wound care). Sample size was determined by the original trial, and the full protocol has been published previously [15].

### Cohort and eligibility criteria

Residents were included if they were using four or more medicines at the time of recruitment, or were taking at least one medicine with anticholinergic or sedative properties. Residents were excluded if they:

A) had significant existing frailty, defined as a score of 0.40 or above using the Frailty Index, [16]
B) had moderate or severe cognitive impairment, measured using the Psychogeriatric Assessment Scales [17] (PAS < 12/21) or Montreal Cognitive Assessment tool (MoCA ≤ 17/30) [18, 19],
C) were receiving palliative care or respite care, or
D) were involved in another research project that affected their participation in this trial.

### Data collection and metrics

Research assistants collected data for all participants at baseline, six months and twelve months. Data included demographics, medical history, Resident Care Assessment, Frailty Index (also called deficit accumulation index) [16], quality of life measured using the EQ5D [20], cognitive function measured using the Montreal Cognitive Assessment (MoCA) test [18] and grip strength.

Grip strength was measured using a handheld dynamometer (Jamar, Illinois, USA) using the dominant hand. The best measurement of three scores was used. To account for the influence of gender in grip strength [21], values were standardised by gender (*z* score).

The anxiety and depression score was taken from the EQ5D, ranging from 1 (“I am not anxious or depressed”) to 5 (“I am extremely anxious or depressed”), which has good performance for screening adults for anxiety and depressive symptoms in community settings [22].

### Operationalisation and data analysis

All frailty domains were operationalised as intrinsic capacity [23]. The operationalised metrics were:

- Multimorbidity: Measured by the number of clinical conditions listed in the Frailty Index
- Muscle strength: Measured by grip strength (standard normalised, stratified by gender)
- Cognitive capacity: measured by MoCA.
- Mood alterations: measured by the anxiety-depression screening question in the EQ-5D-5 levels, given a value 1 for the answer “I am not anxious or depressed” and 5 to “I am extremely anxious or depressed”
- Functional capacity, measured by the limitations to perform activities of daily living (ADL) listed in the Frailty Index

To measure activities of daily living, we created a score based on 16 disability-based questions from the Frailty Index. To measure the number of clinical conditions, we considered 21 of the morbidity related questions in the frailty index. Both scores were calculated in the same way, by summing 1 for each deficit and dividing by the number of valid answers. Therefore, bigger numbers represent more clinical conditions and more limitations to perform activities of daily living. The list of questions and their classification is available at the Appendix 1. All metrics were measured at baseline and repeated at 6 and 12 months. Only individuals alive at 12 months were considered for the analysis.

The primary purpose of the analysis was to systematically identify the relationship between multimorbidity, muscle strength, cognitive capacity, mood and functional capacity over time. To that end, the study will answer three questions: (1) Are the metrics correlated at a population level? The between-individuals metric correlation at baseline was assessed using the Pearson correlation coefficient. (2) Are the metrics predictive of changes at 12 months, at a population level? To answer this question, we fitted a separate Generalised Least Squares linear regression for each of the five metrics as independent predictor and for each of the four remaining metrics as dependent outcome (20 models in total). The Generalised Least Square method was chosen due to the breach of the equality of variance assumption (Levene test result of 21.8, *p* < 0.01). (3) Are metrics correlated within individual. To analyse the interaction between metrics over time, we performed repeated measures correlation [24], which evaluates the association between metrics independently of their association in the population (e.g. whether decline in physical capacity is associated with cognitive decline, rather than whether individuals with poor physical capacity tend to have greater physical decline).

Summary statistics are presented as means, standard deviation and proportions. To account for multiple tests at baseline, we considered a 99% confidence interval (*p* < = 0.01). For all remaining hypotheses, we considered a 95% confidence interval (*p* < = 0.05). All analysis was performed in Python 3.7. We used Python packages Pandas [25], and Scipy [26] for data pre-processing and statistical analysis, respectively. The main statistical library used were Statsmodels [27] and Pingouin [28].

## Results

### Baseline

From August 2018 and July 2020, 248 aged-care residents were recruited, and 208 were alive at the 12-month evaluation. The average age was 85.5 years, average length of stay of 815 days and two-thirds of the participants were women (68%). Baseline metrics can be seen in Table 1. Female participants were older than male participants but had similar frailty index and MoCA scores.

**Table 1:** Baseline summary of enrolled participants alive at the 12-month evaluation

	Grouped by gender		
	Overall	Female	Male
*N* (%)	208	141 (67.8)	67 (32.2)
Age, mean (SD)	85.5 (7.5)	86.1 (7.2)	84.3 (8.1)
MoCA, mean (SD)	22.5 (3.3)	22.3 (3.3)	23.0 (3.4)
Highest grip, mean (SD)	17.5 (7.7)	14.4 (5.4)	23.9 (7.9)
EQ5D_value, mean (SD)	0.7 (0.2)	0.7 (0.2)	0.6 (0.3)
Anxiety/depression score	1.7 (0.9)	1.8 (0.9)	1.7 (0.9)
Morbidity score	0.23 (0.09)	0.23 (0.09)	0.23 (0.09)
Activities of daily living limitation score	0.30 (0.14)	0.30 (0.15)	0.32 (0.14)

Systematic Pearson correlation tests show there was limited overlap between different dimensions at baseline. There were no statistically significant (*p* < 0.01) correlations at baseline.

### Association between baseline measure and future change

At 12-month follow-up, there was significant decline in MoCA (− 2.6 SD 5.47, or 12%), activities of daily living (0.17 SD 0.18, or 52%) and increase in morbidity (0.03, SD 0.06, or 14%) in the studied population. There was a slight increase in grip strength scores (0.18 SD 4.3, or 1%) and slight decrease in anxiety/depression scores (− 0.01, SD 1.03, or 0.7%).

**Fig. 1:**
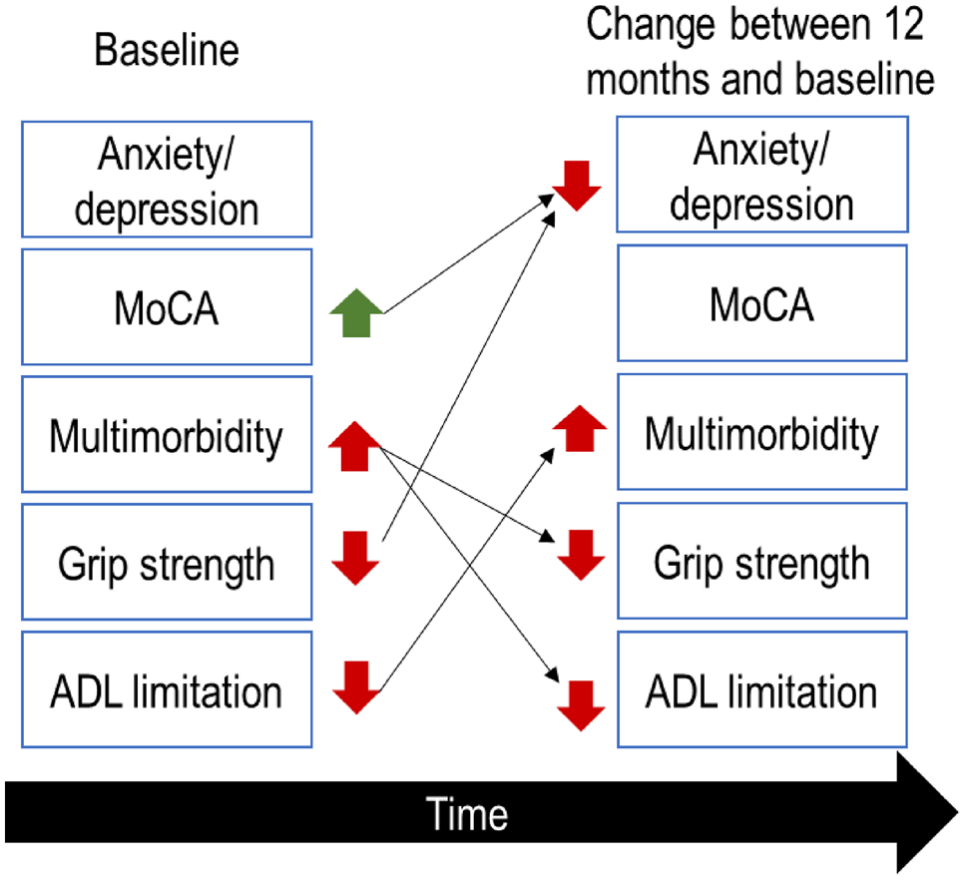
Relationship between frailty-related metrics and future change. The full results table can be found in the supplementary files (Appendix 2)

All of the studied metrics at baseline were associated with change in 12 months, with the exception of anxiety/depression scores. Better MoCA (coef = 0.05, *p* = 0.02) and decreased grip strength (coef = − 0.14, *p* = 0.03) at baseline were associated with worsening of depression scores at 12 months. High morbidity count was associated with a future decrease in capacity to perform daily living activities (coef = 0.4, *p* < 0.01). Similarly, limited capacity to perform daily living activities at baseline was associated with future increase in morbidity count (coef = 0.09, *p* < 0.01). High morbidity count at baseline was associated with future decrease in grip strength (coef = − 1.29, *p* = 0.02). No metric at baseline was associated with changes in MoCA. Figure 1 shows a summary of the associations between baseline and 1-year change, and the full results table listing all five models (one for each dimension as an outcome) can be found in the supplementary files (Appendix 2).

To check for potential biases introduced by the intervention, this analysis was repeated and stratified by group (Appendix 2). Even though none of the associations retained statistical significance in both stratified groups, the general direction of change remained consistent in all groups (intervention, control and all combined).

### Within individual analysis

**Fig. 2:**
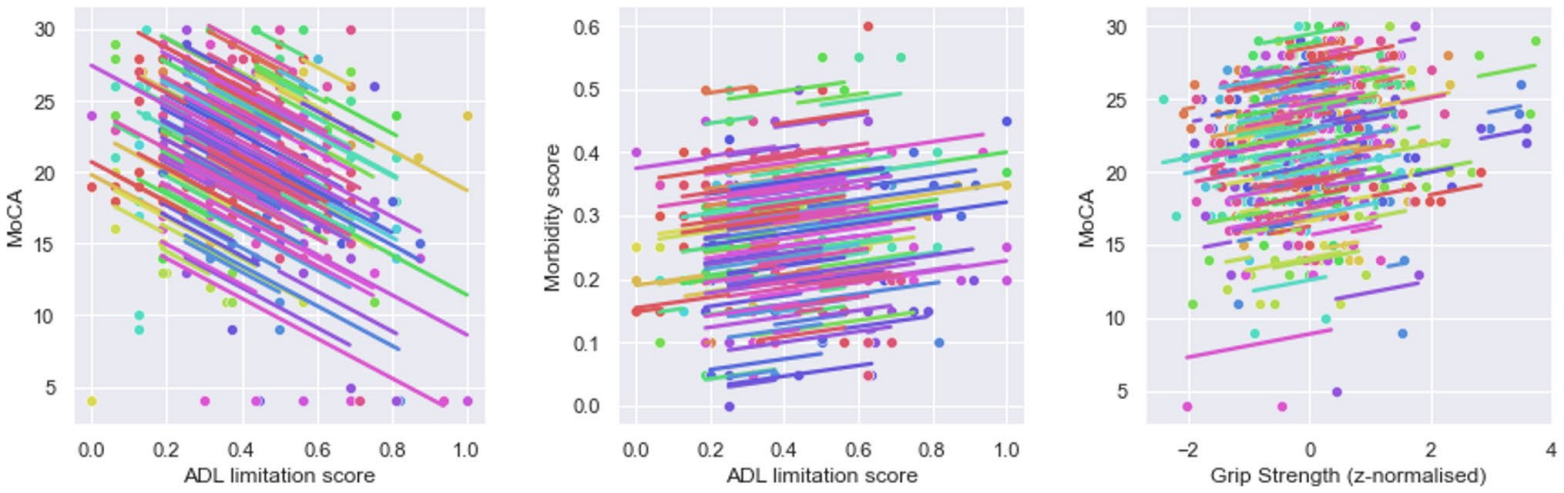
Repeated correlation plot between **a** MoCA and activities of daily living limitation score; **b** Multimorbidity and activities of daily living limitation score; **c** MoCA and normalised grip strength. Each colour represents different individuals, the dots the measures at baseline, 6 months and 12 months, and the parallel lines represent a repeated measure correlation fit for each participant

Pairwise within-individual correlation was statistically significant between MoCA and grip strength (r 0.13, *p* = 0.02) and activity of daily living (r − 0.48, *p* < 0.001), and between activities of daily living and multimorbidity index (r 0.28, *p* < 0.001). No within-individual correlation between anxiety depression score and other metrics was statistically significant. Figure 2 shows the repeated measure correlation plot of the three strongest correlations.

## Discussion

This study tracked five metrics, cognition, muscle strength, mood alterations, multimorbidity and functional capacity, which represent different aspects of frailty over a 1-year follow-up of aged care residents. The results suggest an interdependence between multimorbidity, muscle strength (as measured by grip strength), cognition (measured by MoCA) and functional capacity (measured as capacity to perform activities of daily living) in this population. Ageing and progressive morbidity affect multiple physiological systems and the results shine some light on the complex interaction of those systems over time in older people. The analysis systematically describe how frailty evolves in this usually frail population. In summary, the study shows that metrics were (1) uncorrelated at baseline, but (2) may predict future decline in other metrics, and (3) are associated within individuals, meaning the decline in one follows a decline in another.

The tracked metrics were independent at baseline. Studies of community living individuals suggest that frailty measures are mostly independent in younger individuals [29, 30]. However, the aged care resident population is typically frail (75% frail and 19% pre-frail) [21], and a stronger correlation between all five metrics was expected. Inclusion and exclusion criteria in our trial purposefully excluded very frail individuals and may explain this finding.

Baseline analysis and the influence on change at 12 months paint a somewhat complex picture, with multiple interactions between tracked metrics. A graphical summary of the relationships is shown in Fig. 1. Two particular interactions are noteworthy. The first is that high morbidity at baseline is associated with future decline in capacity to perform activities of daily living, and low capacity for activities of daily living is associated with future increase in morbidity score. This is potentially a self-reinforcing relationship, which may explain the late-life acceleration in frailty [14] as measured by the Frailty Index, a metric composed of both morbidity and activities of daily living scores [31]. The second noteworthy relationship is the association between higher cognitive function at baseline and future worsening of mood alterations (measured by EQ5D anxiety/depression scores). The explanation is not evident, as the link between depression following cognitive decline is well established [32]. Moreover, the lack of population decline of anxiety/depression scores at 12 month follow up is surprising, given the association between frailty scores and neuropsychiatric measures in the aged care population [33]. The results may reflect a shortcoming of EQ5D’s anxiety/depression score as a metric for long term follow up. The EQ5D has a worse performance as a screening tool in hospital settings than community settings and is less sensitive than build for purpose tools such as the GAD for anxiety and PHQ for depression [22]. Besides the potential lack of metric robustness, it is possible that the link between depression and cognition is only valid after a certain threshold and was removed by the eligibility criteria, which excluded participants with dementia and important cognitive decline (MoCA < = 17).

Finally, the study shows a strong within-individual association between all metrics but anxiety/depression score. Taken together, the results suggest that while the metrics are measuring different things (no correlation at baseline), they may trigger further decline and tend to converge over time. These findings are coherent with the systems theory of frailty [34], which states that failure in one node of the network (in this case, one of the frailty metrics) increases damage to other nodes over time.

There are two practical implications of our findings. The first is supporting the concept of frailty as a dynamic decline in multiple physiological systems. The deficit accumulation approach posits that frailty is the accumulation of abnormal clinical features, such as cardiovascular and neurological diseases, leading to a state of decreased functional capacity and susceptibility to additional diseases [35]. This approach could also consider the dynamic [36], continuous and interdependent nature of the frailty process [9, 10]. The second implication is supporting change as a frailty measure. While this study did not include health outcomes, it shows the concurrent decline in multiple systems. It is possible that the rate of change in frailty itself is to be regarded as a clinical problem, as some studies suggest a rapid increase in frailty is associated with poor outcomes [37]. Other studies suggest that change may even surpass frailty score absolute values as a determinant of poor health outcomes [38], although results are equivocal [11]. The results presented here suggest that change is more likely to reflect a global decline in other physiological systems.

Overall, the data presented here suggest frailty is better described as a multidimensional concept over time than as a True/False statement. Frailty scales are a proxy of how well the different physiological systems are working, and may be more strongly associated with self-reported frailty than actual performance decline [39, 40]. However, converging metrics suggest there is an underlying relationship that ties those concepts together over time. Whether periodical measuring of functional capacity by itself is enough to identify potentially frail individuals is subject to future research [41].

The main strength of our study is the longitudinal follow up of older people. As residents in an aged care residence, some degree of impairment was present in all individuals. This cohort will benefit from improved frailty identification measures, for preventative and therapeutic measures to reverse frailty. The main limitation of our study is that it is a sub-study of an interventional clinical trial. The intervention may have altered the natural frailty course and introduced biases. To account for biases, we performed the analysis stratified by intervention or control group. While statistical significance was lost, the direction of associations was consistent in both groups. The relatively small sample size reduced the capacity for further stratification, such as by gender and age, and these variables should be included in future studies. The analysis stratified by intervention group can be found in the appendices. Enrolment criteria may also have introduced a bias which limits generalisability. Recruitment was exclusive for aged care residents but excluded participants with low MoCA and high frailty index. Since this is a population unable to live independently, there may be other factor not measured contributing to increased frailty over time. A cap effect in frailty index (both morbidity and function) meant people with higher frailty index (frail) were less likely to become frailer than individuals with low frailty index score (healthier). Finally, some measures might be inadequate as long term measures of patient function and require further studies with more detailed data collection.

## Conclusion

The results suggest an interdependence between comorbidities, physical capacity, cognition and activities of daily living in aged care residents. Comprehensive repeated measurement of frailty-related metrics may provide improved understanding of frailty progression at later life stages.

### Supplementary Information

Below is the link to the electronic supplementary material.

Supplementary file1 (DOCX 33 KB)

Supplementary file2 (DOCX 25 KB)

## Data Availability

Data available: Yes. Data types: De-identified participant data. How to access data: Please contact the Principal Investigator. When available: With publication. Who can access the data: Requests for data sharing will be reviewed and approved by the data custodian by request. Types of analyses: Requests for data sharing will be reviewed by the data custodian. Mechanisms of data availability: The de-identified dataset will be provided to the requestor in an electronic format.
