# Supplementary material for: Correlation of frailty assessment metrics in one-year follow-up of aged care residents: a sub-study of a randomised controlled trial
Source: Aging Clin Exp Res. 2023 Jul 14;35(10):2081–7. doi: 10.1007/s40520-023-02491-y (PMC10520153; doi:10.1007/s40520-023-02491-y)
Supplement: Supplementary file 2 — Supplementary file2 (DOCX 25 KB) [file 40520_2023_2491_MOESM2_ESM.docx]

APPENDIX 1 - **List of 39 variables included to construct the Frailty Index**

| **Frailty index** | **Scoring** |
| --- | --- |
| **General component** |  |
| Live alone | Yes = 1; No = 0 |
| Self-rated health | - Poor = 1 - Fair = 0.75 - Good = 0.5 - Very good = 0.25 - Excellent = 0 |
| **Comorbidity component** |  |
| Arthritis | Yes = 1; No = 0 |
| Asthma | Yes = 1; No = 0 |
| History of heart attack | Yes = 1; No = 0 |
| Hypertension | Yes = 1; No = 0 |
| Migraine | Yes = 1; No = 0 |
| Parkinson’s disease | Yes = 1; No = 0 |
| History of stroke | Yes = 1; No = 0 |
| Thyroid disease | Yes = 1; No = 0 |
| Ear, nose, throat problem | Yes = 1; No = 0 |
| Mental disorder | Yes = 1; No = 0 |
| Genito-urinary problem | Yes = 1; No = 0 |
| Diabetes | Yes = 1; No = 0 |
| Cancer | Yes = 1; No = 0 |
| Chest pain | Yes = 1; No = 0 |
| Constipation | Yes = 1; No = 0 |
| Dental problem | Yes = 1; No = 0 |
| Sleep problem | - Rarely/never = 0 - Sometimes = 0.5 - Often/almost always = 1 |
| Spinal problem | Yes = 1; No = 0 |
| Hearing difficulty | Yes = 1; No = 0 |
| Eye trouble | Yes = 1; No = 0 |
| Skin problem | Yes = 1; No = 0 |
| **Activities of daily living component** |  |
| Hands shaking problem | - No difficulty at all or a little difficulty = 0 - Some/ a lot of difficulty or just unable to do = 1 |
| Stooping/crouching/kneeling problem | - No difficulty at all or a little difficulty = 0 - Some/ a lot of difficulty or just unable to do = 1 |
| Difficulty with bathing | Yes = 1; No = 0 |
| Difficulty with personal grooming | Yes = 1; No = 0 |
| Difficulty with dressing | Yes = 1; No = 0 |
| Difficulty with eating | Yes = 1; No = 0 |
| Difficulty with toileting | Yes = 1; No = 0 |
| Difficulty with going out | Yes = 1; No = 0 |
| Difficulty with moving around | Yes = 1; No = 0 |
| Difficulty with laundry/linen | Yes = 1; No = 0 |
| Difficulty with housework | Yes = 1; No = 0 |
| Difficulty with preparing meal | Yes = 1; No = 0 |
| Difficulty with using telephone | Yes = 1; No = 0 |
| Difficulty with managing money | Yes = 1; No = 0 |
| Difficulty with using public transport | Yes = 1; No = 0 |
| Difficulty with shopping | Yes = 1; No = 0 |

APPENDIX 2 – Association between baseline metrics and change in 12 months, stratified by intervention group. Each column contains the result of a different model, with changes in 12 months as outcome and baseline values (rows) as determinants.

| All participants (n=208) | Outcome (12-month metric) | | | | | | | | | |
| --- | --- | --- | --- | --- | --- | --- | --- | --- | --- | --- |
|  | MoCA | | Anxiety / Depression | | Grip Strength | | Morbidity | | ADL limitations | |
| Determinants at baseline | coef | p-value | coef | p-value | coef | p-value | coef | p-value | coef | p-value |
| Anxiety / Depression | -0.27 | 0.54 | -0.56 | <0.001* | -0.01 | 0.83 | 0.003 | 0.49 | 0.01 | 0.42 |
| ADL limitations | -0.01 | 1.00 | -0.08 | 0.85 | -0.63 | 0.09 | 0.09 | <0.001* | -0.20 | 0.02* |
| Morbidity | -4.34 | 0.32 | 0.95 | 0.19 | -1.29 | 0.02* | -0.11 | 0.02* | 0.40 | <0.001* |
| MoCA | -0.04 | 0.72 | 0.05 | 0.02* | 0.02 | 0.18 | 0.001 | 0.42 | 0.004 | 0.21 |
| Grip Strength | 0.14 | 0.72 | -0.14 | 0.03* | -0.22 | <0.001* | 0.002 | 0.58 | -0.01 | 0.28 |

* p<0.05

| Intervention group (n=97) | Outcome (12-month metric) | | | | | | | | | |
| --- | --- | --- | --- | --- | --- | --- | --- | --- | --- | --- |
|  | MoCA | | Anxiety / Depression | | Grip Strength | | Morbidity | | ADL limitations | |
| Determinants at baseline | coef | p-value | coef | p-value | coef | p-value | coef | p-value | coef | p-value |
| Anxiety / Depression | -0.03 | 0.96 | -0.69 | <0.001* | 0.06 | 0.59 | 0.01 | 0.38 | 0.03 | 0.23 |
| ADL limitations | -2.79 | 0.52 | -0.16 | 0.83 | -0.18 | 0.78 | 0.08 | 0.04* | -0.16 | 0.23 |
| Morbidity | -4.83 | 0.43 | 1.94 | 0.07 | -1.08 | 0.23 | -0.06 | 0.30 | 0.32 | 0.11 |
| MoCA | 0.03 | 0.84 | 0.04 | 0.13 | 0.03 | 0.26 | 0.002 | 0.13 | 0.004 | 0.43 |
| Grip Strength | -0.10 | 0.86 | -0.16 | 0.10 | -0.20 | 0.02* | 0.004 | 0.52 | -0.01 | 0.63 |

* p<0.05

| Control group (n=111) | Outcome (12-month metric) | | | | | | | | | |
| --- | --- | --- | --- | --- | --- | --- | --- | --- | --- | --- |
|  | MoCA | | Anxiety / Depression | | Grip Strength | | Morbidity | | ADL limitations | |
| Determinants at baseline | coef | p-value | coef | p-value | coef | p-value | coef | p-value | coef | p-value |
| Anxiety / Depression | -0.21 | 0.71 | -0.52 | <0.001* | -0.04 | 0.52 | 0.002 | 0.77 | 0.002 | 0.90 |
| ADL limitations | 1.53 | 0.69 | -0.09 | 0.88 | -0.89 | 0.05* | 0.08 | 0.07 | -0.22 | 0.05* |
| Morbidity | -3.42 | 0.59 | 0.09 | 0.93 | -1.38 | 0.07 | -0.15 | 0.04* | 0.47 | 0.01* |
| MoCA | -0.14 | 0.44 | 0.05 | 0.09 | 0.02 | 0.47 | 0.004 | 0.08 | 0.004 | 0.39 |
| Grip Strength | 0.40 | 0.49 | -0.13 | 0.15 | -0.22 | <0.001* | 0.001 | 0.93 | -0.02 | 0.35 |

* p<0.05
